# Formulation of Multicomponent Chrysin-Hydroxy Propyl β Cyclodextrin-Poloxamer Inclusion Complex Using Spray Dry Method: Physicochemical Characterization to Cell Viability Assessment

**DOI:** 10.3390/ph15121525

**Published:** 2022-12-08

**Authors:** Syed Sarim Imam, Sultan Alshehri, Wael A. Mahdi, Ahmed M. Alotaibi, Moath H. Alhwaifi, Afzal Hussain, Mohammad A. Altamimi, Wajhul Qamar

**Affiliations:** 1 Department of Pharmaceutics, College of Pharmacy, King Saud University, Riyadh 11451, Saudi Arabia; 2 Department of Pharmacology and Toxicology, College of Pharmacy, King Saud University, Riyadh 11451, Saudi Arabia

**Keywords:** chrysin, HP βCD, poloxamer, antimicrobial, solubility, cell viability

## Abstract

The work aimed to enhance chrysin (CHR) water solubility, dissolution, and in vitro antibacterial as well as cell viability. Chrysin binary, as well as ternary inclusion complex, were prepared using the spray drying method. The influence of an auxiliary component (poloxamer; PLX) was also assessed after being incorporated into the chrysin HP βCD complex (CHR-BC) and formed as a chrysin ternary complex (CHR-TC). The phase solubility investigation was carried out in order to assess the complexation efficiency and stability constant. The samples were assessed for the dissolution test, physicochemical evaluation, antibacterial activity, and cell viability tests were also assessed. The results of the phase solubility investigation showed that the stability constant for the binary system (268 M^−1^) was lower than the ternary system (720 M^−1^). The complex stability was validated by the greater stability constant value. The dissolution results showed that pure CHR had a limited release of 32.55 ± 1.7% in 60 min, while prepared CHR-TC and CHR-BC both demonstrated maximum CHR releases of 99.03 ± 2.34% and 71.95 ±2.1%, respectively. The dissolution study’s findings revealed that the release of CHR was much improved over that of pure CHR. A study using a scanning electron microscope showed that CHR-TC contains more agglomerated and amorphous components. The higher conversion of crystalline CHR into an amorphous form is responsible for the structural alterations that are observed. After complexation, the distinctive peaks of pure CHR changed due to the complexation with HP βCD and PLX. The antimicrobial and cell viability results revealed improved antimicrobial activity as well as a lower IC50 value than pure CHR against the tested anticancer cell line (MCF7).

## 1. Introduction

A dietary flavonoid called chyrsin (CHR) is abundantly found in various natural resources such as honey, propolis, honeycomb, and blue passionflowers [1,2]. It is used as a food supplement without any side effects [3,4]. Similar to other flavonoids, it exhibits a wide range of pharmacological activities such as antioxidant, anticancer, anti-inflammatory, and antihypertensive [5]. It has shown potential biological and nutritional effects with lesser systemic toxicity. It is reported as a poorly water-soluble drug which limits its bioavailability [6,7]. Different CDs are used to improve the solubility of poorly soluble drugs. Through its interaction with various hydrophobic guest molecules in an aqueous solution, it produces an inclusion complex [8,9].

In comparison to a pure or uncomplexed molecule, the solubility is significantly increased by the formed inclusion complex. It can improve the drug solubility and is found stable in super-saturated solutions [10]. The chemical alteration of β CD has emerged as an important technique to enhance water solubility. In addition to increasing β CD water solubility, the chemical modification also allows for the adjustment of the hydrophobic cavity’s spatial shape to accommodate the drugs. In order to regulate the physicochemical characteristics of β CD, hydroxyl groups were subjected to chemical transformations such as esterification, etherification, and hydroxyl-alkylation [8]. Hydroxypropyl-cyclodextrin (HP βCD) is a biocompatible and biologically inert molecule. It has been widely used in a number of formulations to enhance water solubility [11]. HP βCD has reported a 50-fold enhancement in solubility as well as low toxicity [12]. Recent study reports have revealed that the inclusion of an auxiliary agent can improve the solubility and complexation efficiency of CDs [13]. It forms co-complexes that may offer higher stability constant [10]. It increases CDs’ solubility power and lowers the number of CDs required to achieve the desired solubilization result [14].

The goal of the current research was to prepare CHR/HP βCD with and without PLX inclusion complex by spray-drying technique. The complexes were evaluated for drug release, physicochemical evaluation, cell viability, and antibacterial activity.

## 2. Results and Discussions

### 2.1. Phase Solubility Assessment

**Figure 1:**
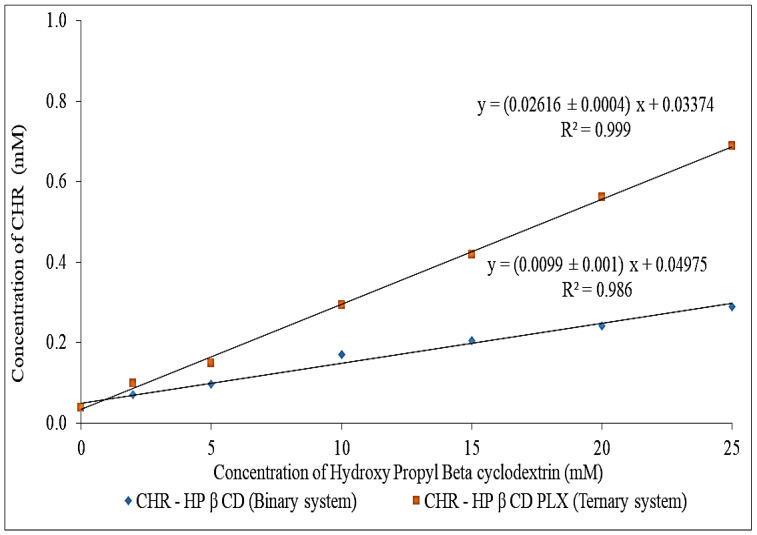
Phase solubility curve of (CHR + HP βCD) and CHR + HP βCD + PLX.

The results depicted that the solubility of CHR was gradually improved with gradual enhancement in HP βCD concentration (Figure 1). The binary, as well as ternary sample graphs, show the A_L_ type of diagram. The complexation efficiency (CE), as well as stability constant (Ks) were calculated, and the binary system showed stability constant (Ks) value of 268 M^−1^. A significantly higher value (720 M^−1^) was found for the ternary system. The higher value of the stability constant confirmed that the complex was found to be stable [15]. A ternary substance is added, which interacts with CDs’ outer surfaces, and drug CD complexes and also helps in the formation of co-complexes or aggregates [16]. A value smaller than 100 m/L forms an unstable drug CD complex, and a value greater than 1000 m/L has a negative impact on drug absorption [17]. In addition to providing a more accurate measurement of the solubilizing efficacy of the complexing agent, it also avoids the risk of utilizing inaccurate determination of the intrinsic solubility of drugs that are poorly soluble [14].

### 2.2. Dissolution Study

**Figure 2:**
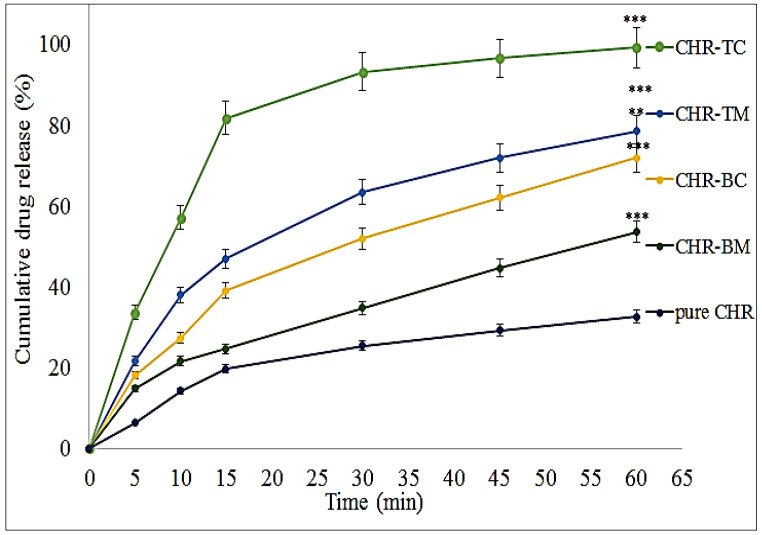
The dissolution profile of pure Chrysin (CHR), Chrysin binary mixture (CHR-BM), Chrysin binary complex (CHR-BC), Chrysin ternary mixture (CHR-TM), and Chrysin ternary complex (CHR-TC). The study was performed thrice, and results were shown as mean ± SD. *** highly significant to pure Chrysin; ** significant to Chrysin ternary mixture.

The dissolution profile depicted a significant (*p* < 0.001) improvement in the CHR release behavior. The CHR-BM, CHR-BC, CHR-TC, and CHR-TM showed better release than pure CHR. The order of release was found to be CHR-TC (99.03 ± 0.34 %) > CHR-TM (78.5 ± 3.1 %) > CHR-BC (71.9 ± 2.1 %) > CHR-BM (53.62 ± 3.1 %) > pure CHR (31.5 ± 0.7 %) in 60 min as shown in Figure 2. CHR-BM and CHR-BC showed significantly (*p* < 0.05) enhanced CHR release in the 60 min. The cumulative CHR release from CHR-BC and CHR-BM was found to be 27.3 ± 2.1% (about 2 fold) and 21.6 ± 2.1% (1 fold) in the first 10 min. The enhancement in CHR solubility was achieved by partial entrapment into the HP βCD [18]. The improved CHR dissolution achieved from BM may be attributed to an enhancement in the drug’s wettability and solubility at the early stages of the dissolution [19]. HP βCD has a hydrophilic surface from the outside, which gives it surfactant-like qualities [16]. Further enhancement in the release was achieved after the addition of PLX to the CHR—HP βCD complex. CHR-TC and CHR-TM depicted significantly (*p* < 0.001) enhanced CHR release. The cumulative CHR release from CHR-TC and CHR-TM was found to be 56.1 ± 1.5% (about 4 fold) and 38.1 ± 1.5% (2 fold) in the first 10 min. The presence of a ternary substance (PLX) may help to achieve greater inclusion, complexation, and amorphization. The elements that contribute to the better and enhanced dissolution efficiency of the drug include the transformation of the drug from a crystalline to an amorphous state, higher hydrophilicity, development of hydrogen bonds as well as wetting properties of the carrier [20]. The study was performed with PLX at 10 % *w*/*w*. It showed maximum solubility by decreasing the drug crystallinity. The dissolution step requires less energy, and the drug is uniformly dispersed [18]. The intrinsic dissolution rate was slower at higher PLX concentrations in the inclusion complex than at lower concentrations. It facilitates the production of gel layers in water, which serve as diffusion barriers and prolong drug release [18,21,22]. A synergistic interaction of two solubilizers (HP CD and PLX) may increase the drug’s solubility. The drug is subjected to the surfactant’s (micellar solubilization activity) and CDs complexation properties as solubilizers, respectively [23]. According to the SEM study, the complexes made by the spray drying technique had a better dissolving profile than the physical mixture due to lesser crystallinity [17].

### 2.3. Particle Characterization

The prepared samples were evaluated for particle size, polydispersibility index, and zeta potential. The results showed that the particle was found in the range of 800–1200 nm. The formulation CHR-TM showed a larger particle size of 1181 ± 4.1 nm, whereas the CHR-TC depicted a smaller size of 941 ± 6.4 nm. The difference in size is due to the difference in the method of preparation. The sample was prepared by a spray dryer having a lesser size than the physical mixture. Monodisperse systems are indicated by values close to zero (< 0.10), while polydisperse systems are indicated by values greater than 0.10 [24]. CHR-TM and CHR-TC showed the PDI value of 0.39 ± 0.012 and 0.26 ± 0.021, as well as zeta potential values of −17.7 ± 3.2 mV and −25.2 ± 4.1 mV. A significant variation in the size, PDI, and ZP was observed between the two samples. The prepared CHR-TC showed higher zeta potential than the CHR-PM. That means it is more stable than the prepared physical mixture. The complexation of CHR with CDs could block these hydroxyl groups since the negative surface of cyclodextrin indicates that the hydroxyl groups are primarily oriented toward the surrounding aqueous media [25].

### 2.4. Surface Morphology

**Figure 3:**
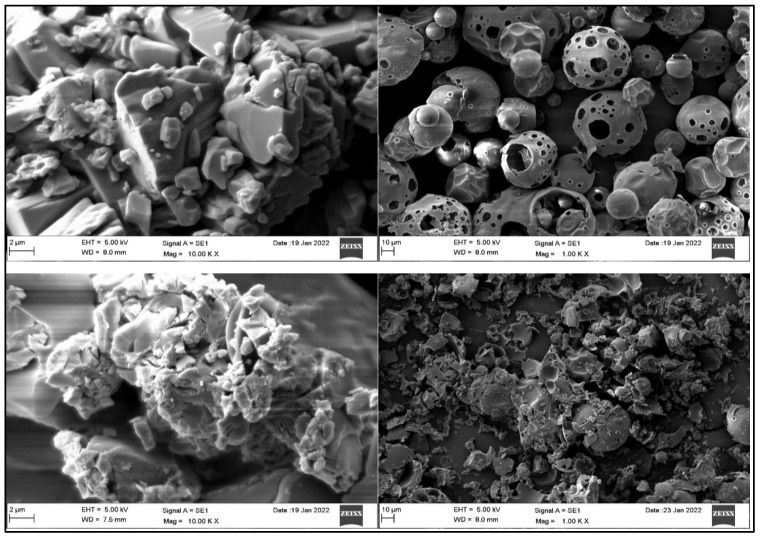
Scanning electron micrograph of pure Chrysin, HP-βCD, Chrysin ternary mixture (CHR-TM), and Chrysin ternary complex (CHR-TC).

Figure 3 depicts the surface morphology of pure CHR, HP βCD, CHR-TM, and CHR-TC to evaluate changes in structure. The pure CHR displayed irregularly shaped particles with no discernable form. The shape of HP βCD is amorphous and indistinct [18]. Fewer crystal structures were found in CHR-TM due to the milling procedure, which reduces the particle size, although some agglomerates were still visible. A greater transition from a crystalline to an amorphous state occurred in the case of CHR-TC, and homogeneous agglomerates with uneven porosity surfaces were evident [26]. The very small size of the particles gave them a greater propensity to penetrate and disperse.

### 2.5. Fourier-Transformed Infrared Spectroscopy

**Figure 4:**
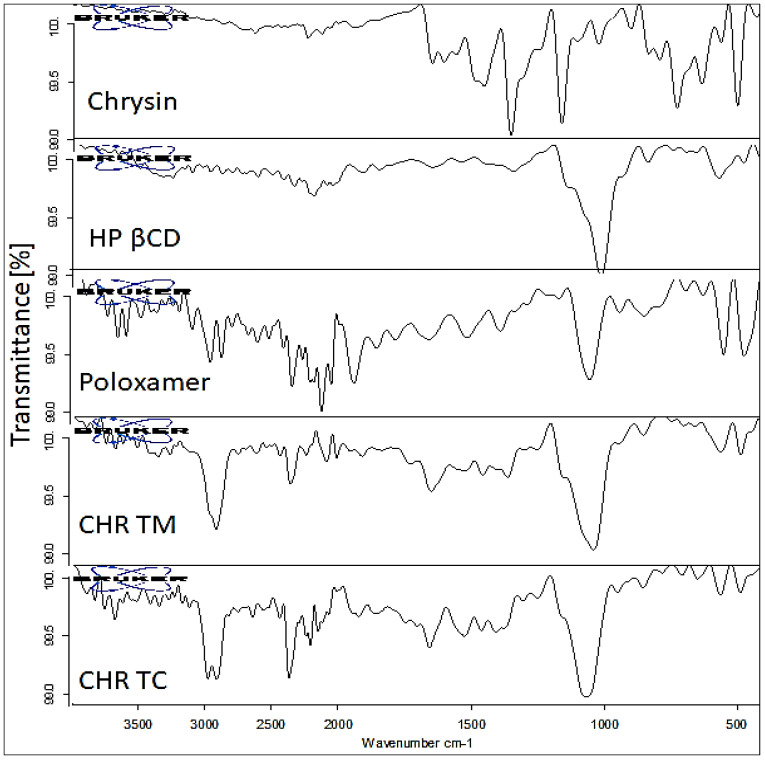
IR spectra of pure CHR, HP βCD, PLX, Chrysin ternary mixture (CHR-TM), and Chrysin ternary complex (CHR-TC).

The allocated frequency at Infrared vibrations for the pure CHR, Poloxamer (PLX), HP βCD, CHR-TM, and CHR-TC are depicted in Figure 4. CHR, known as 5,7-dihydroxyflavone, depicted a sharp hydroxyl stretching vibration at 3261 cm^−1^, carbonyl (C=O) peak at 1603 cm^−1,^ and pyran ring peak at 1454 cm^−1^, respectively. The carrier HP βCD was recognized at 3260 and 1021 cm^−1^, which corresponds to the stretching vibration of O–H and C-O-C moiety. Whereas PLX showed characteristic peaks at 3499 cm^–1^ (O–H stretching) and 1064 cm^–1^ for C–O-C stretching. The physical mixture (CHR-TM) showed peaks of the carrier at 3260 cm^–1^ for hydroxyl (-OH) stretching and carbonyl (C=O) stretching vibration at 1526 cm^−1^. It has also been noticed that there was a slight change in peaks for the pyran ring at 1458 cm^−1^ as compared to pure CHR. In the case of CHR-TC, a marginal change in CHR peaks of the functional groups viz., hydroxyl, carbonyl, and pyran ring was observed. The functional groups of the PLX and HP βCD exhibited a drastic change in their characteristics, peaking at 3398 cm^–1^ for O–H stretching. The C-O-C stretching vibration peaks were observed at 1064 cm^–1^. The above spectral changes in the characteristic peaks confirm the formation of the inclusion complex.

### 2.6. Nuclear Magnetic Resonance

**Figure 5:**
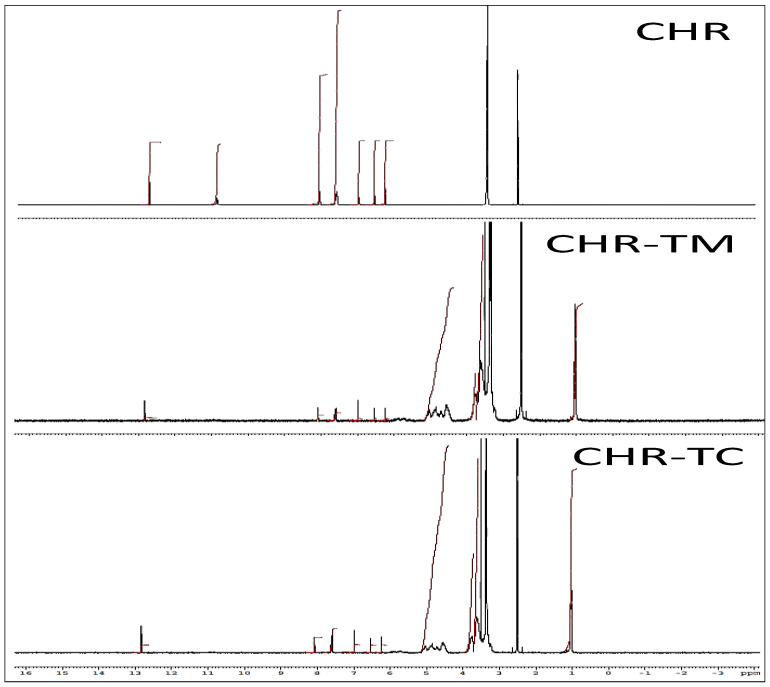
Spectra of pure Chrysin (CHR), Chrysin ternary mixture (CHR-TM), and Chrysin ternary complex (CHR-TC).

NMR of pure CHR, PLX, HP βCD, CHR-TM, and CHR-TC was elucidated to visualize the formation of the complex (Figure 5). This NMR characterization will further strengthen the findings of IR spectroscopy. The proton NMR spectrum of CHR depicted a deshielded singlet peak at 12.82 and 10.93 ppm, which is attributed to the C-5 and C-7 OH proton of flavonoids. The aromatic carbon showed multiple peaks at 6.23–7.61 ppm for the pure CHR. The pyran ring exhibited a singlet peak at δ 8.07 ppm. The carrier PLX showed a distinct deshielded singlet peak at 3.35 ppm, ascribed to the methylene hydroxy group, which is concealed by the water signal in the spectra. The singlet peak for the carrier HP βCD was scouted at 5.39 ppm at position 1 of the glucose moiety. The singlet peak of the hydroxyl moiety of HP βCD was depicted at δ 2.63 ppm. The physical mixture (CHR-TM) depicted a distinct deshielded singlet at 12.83 ppm, which is attributed to the C-5 hydroxyl of the flavonoid aromatic ring. The C-7 OH proton of the flavonoid aromatic ring disappeared in the CHR-TM. The aromatic carbon showed multiple peaks at 6.23–7.63 ppm of the pure drug. It also exhibited a pyran ring definite singlet peak at δ 8.09 ppm. A distinct deshielded singlet peak of the carrier, with a noticeable change at 3.61 ppm, depicts the OH proton of the carrier. The prepared CHR-TC exhibited the ^1^H NMR spectrum of CHR, a distinct deshielded singlet at 12.83 ppm, which is remarked to the C-5 hydroxyl proton of the flavonoid aromatic ring. The C-7 OH proton of the pure drug was also absent in the prepared formulation. The aromatic carbon showed multiple peaks of the pure CHR at 6.23–7.61 ppm, which is in agreement with the pure drug. CHR-TC also showed the peaks for PLX at a distinct deshielded singlet (3.75 ppm) which is accredited to the hydroxyl proton of the carrier. Apart from the above observations in the chemical shift, there were other peaks that were discovered during proton spectroscopy in the range of 4–6 ppm, which showed the CHR with the carriers, exhibiting the formation of the complex.

### 2.7. Antimicrobial Activity

**Figure 6:**
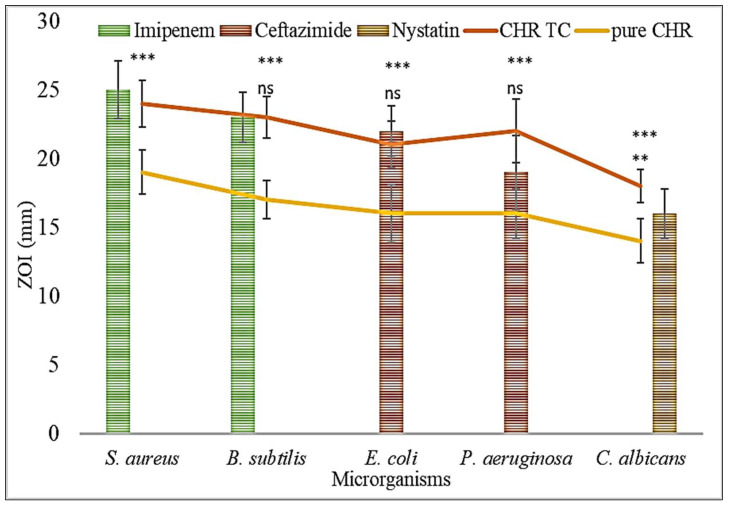
Antimicrobial profile of pure Chrysin (CHR) and Chrysin ternary complex (CHR-TC), and standard compound (Imipenem, Ceftazimide, and Nystatin). The study was performed three times, and the results were shown as mean ± SD. *** highly significant to pure Chrysin, ** significant to pure Chrysin; ns—nonsignificant.

CHR has been reported for its antibacterial activity [27]. It acts by destroying the integrity of the microbial cell wall and cell membrane [28]. The comparative antibacterial study results of the pure CHR and CHR-TC with standard compounds were assessed against various microorganisms (Figure 6). The pure CHR shown ZOI against *S. aureus* (19 ± 1.6 mm), *B. subtilis* (17 ± 2.2 mm), *E. coli* (16 ± 1.9 mm), *P. aeruginosa* (16 ± 2.1 mm), and *C. albicans* (14 ±1.8 mm). Due to the significant improvement in solubility, the prepared CHR-TC demonstrated improved antibacterial efficacy against all the tested organisms. CHR-TC showed the ZOI values against the tested organisms as *S. aureus* (24 ±2.3 mm), *B. subtilis* (23 ± 1.9 mm), *E. coli* (21 ±1.8 mm), *P. aeruginosa* (22 ±3.2 mm), and *C. albicans* (18 ± 2.1 mm). The cytoplasmic membrane and cell wall may be destroyed as a result of the aforementioned findings, which may be caused by the greater solubility of the CHR inclusion complex. There was leakage of cytoplasmic content of microbes, which led to higher destruction of bacteria [29,30]. The standard drug also depicted high ZOI against the tested organisms, and the ZOI of CHR-TC was found to be closer.

### 2.8. Cell Viability

**Figure 7:**
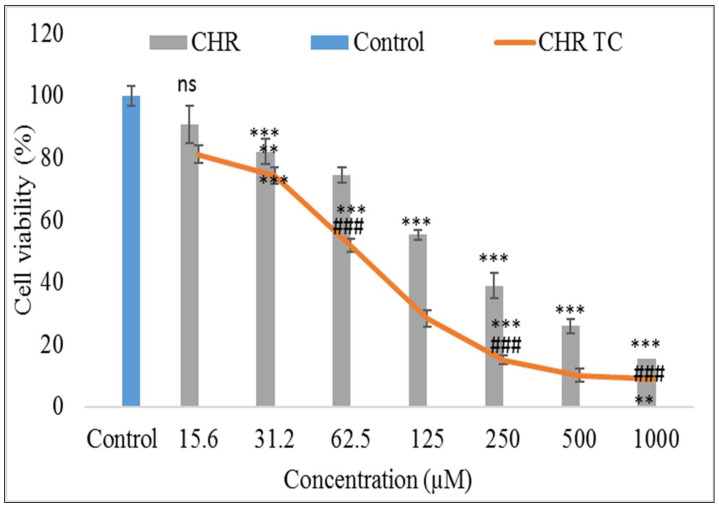
Cell viability study of pure Chrysin (CHR) and Chrysin ternary complex (CHR-TC) against breast cancer cell line (MCF-7). The study was performed three times, and data was shown as mean ± SD. *** highly significant to control; ** significant to pure Chrysin; ### highly significant to pure Chrysin; ns—nonsignificant.

The cell viability of the CHR-TC and pure CHR has been evaluated against the breast cancer cell (MCF7). The study showed a significant (*p* < 0.0001) effect from pure CHR and CHR-TC in the concentration of 15.6 µM to 1000 µM, compared to the control (Figure 7). The pure CHR showed lower cell viability (%) at all concentrations than CHR-TC. At initial concentration, both pure CHR and CHR-TC showed less activity. At initial test concentration, the pure CHR showed less activity at 15.6 µM (90.8 %) and 31.5 µM (82.1 %) than the CHR-TC at 15.6 µM (81.2 %) and 31.5 µM (74.3 %). The difference was found to be slightly significant. As the concentration of CHR increases, the effect was found to be extremely significant (*p* < 0.001). The higher activity is shown by the pure CHR. It showed highly significant activity at the concentrations of 62.5 µM (74.6 %), 125 µM (55.36 %), 250 µM (39 %), 500 µM (26%), and 1000 µM (15.5 %). About 50 % of the cells were viable at 125 µM concentration, and further activity was increased. However, in the case of CHR-TC, the activity was found to be significantly (*p* < 0.01) higher at all the concentrations. It was more effective at concentrations of 62.5 M (51.9%), 125 M (28.1%), 250 M (15.1%), 500 M (10.1%), and 1000 M (9.1%). It showed 50 % viability at a 2-fold lower concentration (62.5 µM), and the difference was found to be highly significant. About 2 to 3-fold higher activity was achieved at 125 µM, 250 µM, and 500 µM. So, it can be concluded that the activity was found to be concentration-dependent. It showed cell toxicity by inhibiting cell proliferation. The inhibition of cell growth was in agreement with the published research reported [31,32]. They used chrysin that had been isolated from Scutellaria plant roots, stems, and leaves. After 4 days of therapy, they reported the activity against the MDA MB 231 cancer cell line at a 100 M concentration. The drug’s immediate inhibitory effect following a specific exposure period must also be evaluated. The IC50 value was also determined, and the difference was found to be significant (*p* < 0.001). The CHR-TC displayed a lower IC50 value (76.94 µM) than the pure CHR, which displayed an IC50 value of 182.53 µM. Our findings are similar to earlier reported research. They reported that CHR has cytotoxic action against cancerous human breast cells and demonstrated it has anti-carcinogenic and anti-tumor characteristics [13,33].

## 3. Materials and Methods

Chrysin was procured from Sigma Aldrich, St. louis, MO, USA. Hydroxypropyl β cyclodextrin (HP βCD) and Poloxamer (PLX) were purchased from Alfa Aesar, Ward Hill, MA. The cancer cell lines lung cancer (A549), and breast cancer (MCF-7) were obtained from the German Collection of Microorganisms and Cell Cultures (DSMZ) (Braunschweig, Germany). Analytical-grade compounds and solvents were employed for the study.

### 3.1. Phase Solubility Assessement

The phase solubility assessment for the binary and ternary samples was performed as per the reported method [34]. An excess quantity of CHR (supersaturation stage) was added to an aqueous HP βCD (0–25 mM) solution (25 mL). The resultant mixture was agitated in a water bath shaker at room temperature (25 °C) for 3 days. Similarly, a ternary sample was prepared with the addition of PLX (10 %, *w*/*w*) to the binary sample. The study was performed in the same condition. After the stipulated time, the sample was taken from the flask, filtered, and diluted (if needed). The amount of CHR present in each HP βCD concentration was determined using a UV spectrophotometer (Shimadzu 1601PC, Kyoto, Japan). From the absorbance, the graph was plotted, and the slope was used to calculate the stability constant (Ks) and complexation efficiency (CE).

(1) $$\mathrm{Ks}=\frac{\mathrm{slope}}{\mathrm{So}\;\left(1-\mathrm{slope}\right)}$$

So = CHR solubility without excipients.

(2) $$\mathrm{CE}=\frac{\mathrm{slope}}{1-\mathrm{slope}}$$

### 3.2. Formulation of Inclusion Complex

The CHR–HP βCD (1:1 moles) and CHR–HP βCD–PLX (1:1 M:10 % *w*/*w*) complex were prepared using the spray dryer method. The ingredients HP βCD/CHR and HP βCD/CHR/PLX were used to prepare binary as well as ternary complex. The complex was prepared by a slightly modified spray drying technique [35]. A spray dryer Buchi B-191 micro (Buchi Labortechnik AG, Flawil, Switzerland) was used to carry out the preparation of the inclusion complex. The study was performed at operational parameters pump of 10%, a flow rate of 600 L/h, an outlet drying temperature of 110 °C, and an aspirator level of 85%. In order to keep the residue in the feed pipe, the solvent was removed and the content re-dissolved in water. For characterization, all the spray-dried complexes were kept in a desiccator. CHR–HP βCD (binary physical mixture) and CHR–HP βCD–PLX (ternary physical mixture) were prepared by thoroughly combining each ingredient. In a mortar and pestle, precisely weighed amounts of the materials were carefully combined and triturated.

### 3.3. Dissolution Study

In order, to assesses the release over a certain period of time, the dissolution study for pure CHR, CHR-BM, CHR-TM, CHR-BC, and CHR-TC was carried out. The study was performed at a temperature of 37 ± 1 °C a rotation speed of 50 rpm with 900 mL dissolution medium (0.1 N HCl) using the apparatus (Distek Dissolution system 2500, Distek, NorthBrunswick, NJ, USA) [36,37]. The samples were added to the release medium, and after certain intervals, 5 mL of released content was collected and replenished with the same volume. CHR content at each time was measured using a UV spectrophotometer after the appropriate dilution [37].

### 3.4. Particle Characterization

The prepared chrysin ternary mixture and chrysin ternary complex (CHR-TM, CHR-TC) were evaluated for particle size, polydispersibility index (PDI), and zeta potential (surface charge). The sample was diluted in double distilled water (10 mL) and filtered using a membrane filter. From this, the solution (0.1 mL) was collected and further diluted. The samples were transferred to a cuvette and analyzed for particle size and PDI using a zetasizer (Malvern ZS90, Malvern, UK). Zeta potential was also evaluated from the same sample using a special electrode containing a cuvette. The values for both CHR-TM and CHR-TC were noted.

### 3.5. Surface Morphology

The surface morphology of the pure CHR, HP βCD, CHR-TM, and CHR-TC was visualized under a high-resolution microscope (Scanning electron microscope; JOEL, Tokyo, Japan). A small quantity of each sample was taken and coated with gold to evaluate under a microscope.

### 3.6. Fourier Transform Infrared Spectroscopy

The characteristic peaks of the pure excipients were compared with the prepared complex formulation. Each sample spectra (CHR, HP βCD, PLX, CHR-TM, and CHR-TC) was compared to one another, and the results were concluded. The alteration in peaks helps to identify the drug-polymer interaction and the formation of the inclusion complex. The IR spectral analysis was performed by ATR-FTIR (Bruker Alpha, Germany). Each sample was run between 4000 and 400 cm^−1^ at a resolution 2 cm^−1^, and spectra were assessed for any conformational changes.

### 3.7. Nuclear Magnetic Resonance

In order to assess the prepared complex physical characteristics and structural changes; an NMR analysis was conducted. The investigation involved comparing the spectra of CHR, PLX, HP βCD, CHR-TM, and CHR-TC. ^1^H NMR (Bruker NMR; Bruker, Switzerland) was used to evaluate these materials. Deuterated DMSO was used for the study, while TMS was used as an internal standard.

### 3.8. Antimicrobial Study

The comparative antimicrobial study was assessed for the pure CHR and CHR-TC in the nutrient broth medium. The bacterial strain was grown to an OD600 of 0.6 for constant bacterial load, which was equivalent to 5.0 × 10^−6^ CFU/mL. The organisms were grown in the nutrient broth to the same optical density. The nutrient agar medium is used to grow in Petri plates for assessing zones of inhibition. The media was incubated at 37 ± 1 °C for 24 h. The nutrient agar media (25 mL) was sterilized and cooled to room temperature, and then the culture suspension (1 mL) was mixed completely. The mixture was poured into Petri plates for solidification, and a well (8 mm in diameter) was created with a sterilized stainless-steel borer. The test samples (pure CHR, CHR-TC, and standard compounds) were added to each well. The plates were covered and kept in the same position for 1 h and further incubated for 24 h to check the effects [38].

### 3.9. Cell Viability Assessement

The breast cancer cell line (MCF7) was used to perform a cell viability study, and the results shown as 50 % inhibition in cell growth (IC_50_). The drug dose caused treated cells to have a 50% lower absorbance than untreated cells. The pure CHR and CHR-TC were tested in vitro for their effectiveness on the breast cancer (MCF7) cell line. CHR-TC and pure CHR results were compared to assess the effectiveness. MTT test (3-(4,5-dimethylthiazol-2-yl)-2,5-diphenyl tetrazolium bromide) was used to analyze the samples, and the results were compared. This test relies on mitochondrial dehydrogenases to transform MTT into formazan crystals and is further utilized to assess the viability of the cells. In 96-well plates of Dulbecco-modified Eagle media (DMEM) (10% Foetal Bovine Serum), the breast cancer cells (MCF7) were cultivated and kept in an incubator with a steady supply of 5% CO_2_ for 24 h. The samples were taken at various doses to measure cell viability. In order to allow cells to metabolize the MTT, the treated samples were incubated for 4 h. The formazan was dissolved in DMSO following the replacement of the media from each well. After 30 min of incubation, the estimation was performed at 570 nm with DMSO acting as a blank. The viable cells (%) were expressed using the formula:

(3) $$\%\;\mathrm{Cell}\;\mathrm{viability}\;=\frac{\mathrm{OD}\;\mathrm{of}\;\mathrm{control}\;\mathrm{cells}}{\mathrm{OD}\;\mathrm{of}\;\mathrm{treated}\;\mathrm{cells}}\times 100$$

### 3.10. Statistical Analysis

The tests were performed in triplicates, and the results are shown as the mean ± SD. The statistics one-way ANOVA was used, followed by Tukey analysis to compare the various samples.

## 4. Conclusions

The two different types of inclusion complexes using CHR with HP βCD (binary) and CHR, HP βCD, and PLX (ternary) complex were prepared using the spray-dried technique. The phase solubility study showed the constant stability value in the acceptable range. The prepared complexes were evaluated for different parameters. There was a noticeable increase in the CHR release achieved from the binary and ternary samples. IR and SEM study results revealed the formation of a complex. The changes in structure are observed due to the conversion of crystalline CHR into an amorphous form. It was further confirmed by the NMR spectral analysis. The antimicrobial activity results revealed better activity than pure CHR due to enhanced solubility. The cell viability results revealed less toxicity against the tested anticancer cell line (MCF7).

## Data Availability

Data is contained within the article.
